# Decomposition of *Fomes fomentarius* fruiting bodies – transition of healthy living fungus into a decayed bacteria-rich habitat is primarily driven by Arthropoda

**DOI:** 10.1093/femsec/fiae044

**Published:** 2024-03-29

**Authors:** Jason Bosch, Priscila Thiago Dobbler, Tomáš Větrovský, Vojtěch Tláskal, Petr Baldrian, Vendula Brabcová

**Affiliations:** Laboratory of Environmental Microbiology, Institute of Microbiology of the Czech Academy of Sciences, 142 00 Prague, Czechia; Laboratory of Environmental Microbiology, Institute of Microbiology of the Czech Academy of Sciences, 142 00 Prague, Czechia; Laboratory of Environmental Microbiology, Institute of Microbiology of the Czech Academy of Sciences, 142 00 Prague, Czechia; Laboratory of Environmental Microbiology, Institute of Microbiology of the Czech Academy of Sciences, 142 00 Prague, Czechia; Laboratory of Environmental Microbiology, Institute of Microbiology of the Czech Academy of Sciences, 142 00 Prague, Czechia; Laboratory of Environmental Microbiology, Institute of Microbiology of the Czech Academy of Sciences, 142 00 Prague, Czechia

**Keywords:** deadwood fungi, *Fomes fomentarius*, fruiting body, microbial communities, mycelium decomposition, temperate forest

## Abstract

*Fomes fomentarius* is a widespread, wood-rotting fungus of temperate, broadleaved forests. Although the fruiting bodies of *F. fomentarius* persist for multiple years, little is known about its associated microbiome or how these recalcitrant structures are ultimately decomposed. Here we used metagenomics and metatranscriptomics to analyse the microbial community associated with healthy living and decomposing *F. fomentarius* fruiting bodies to assess the functional potential of the fruiting body-associated microbiome and to determine the main players involved in fruiting body decomposition. *F. fomentarius* sequences in the metagenomes were replaced by bacterial sequences as the fruiting body decomposed. Most CAZymes expressed in decomposing fruiting bodies targeted components of the fungal cell wall with almost all chitin-targeting sequences, plus a high proportion of beta-glucan-targeting sequences, belonging to Arthropoda. We suggest that decomposing fruiting bodies of *F. fomentarius* represent a habitat rich in bacteria, while its decomposition is primarily driven by Arthropoda. Decomposing fruiting bodies thus represent a specific habitat supporting both microorganisms and microfauna.

## Teaser

An analysis of the metagenomics and metatranscriptomics of healthy living and rotten *Fomes fomentarius* fruiting bodies revealed strong dominance of bacteria in the metagenome of decomposing fruiting bodies, however, numerous transcripts involved in chitin decomposition indicate involvement of Arthropoda in fungal fruiting body decomposition.

## Introduction

*Fomes fomentarius* is a basidiomycete from the family Polyporaceae and a common deadwood-decomposing fungus throughout the Northern Hemisphere forests, preferentially colonizing European beech (*Fagus sylvatica*) and silver birch (*Betula pendula*) wood (Schwarze 1994, Müller et al. 2007, Kacprzyk et al. 2014). It is an effective white rot decomposer and an important contributor to forest carbon and nitrogen cycling. As a white rot fungus, *F. fomentarius* is capable of digesting all the major components of deadwood (lignin, cellulose and hemicellulose) resulting in more complete decomposition of the deadwood and preparing the deadwood for organisms which are unable to digest lignin (Fukasawa 2021). *Fomes fomentarius* is both a frequent parasite of living trees and an early coloniser of deadwood. As the latter, it has a stimulatory effect on deadwood colonization by many other wood-decay fungi, influencing the development of the subsequent microbial community (Heilmann-Clausen and Boddy 2005).

While the main role of *F. fomentarius* is deadwood decomposition, the fruiting bodies also provide other ecosystem services. Living sporocarps may serve as a habitat for other taxa, are known to be associated with deadwood-inhabiting insects (Thunes 1994, Økland 2002, Friess et al. 2019, Seibold et al. 2019) and co-occur with bark beetles. Bark beetles may have devastating effects on forests (Štursová et al. 2014) and are potentially all mycophagous at some point in their life cycle (Harrington 2005). Fungal fruiting bodies have been demonstrated to host specific bacterial communities that differ among fungal species (Pent et al. 2017, Liu et al. 2018, Gohar et al. 2020, Pent et al. 2020). However, no studies have specifically addressed the microbiome of wood-rotting fungi, including the fruiting bodies of *F. fomentarius* which can live for up to 25 years (Thunes 1994).

After death, fungi continue to influence their associated communities. Thunes and Willassen (1997) identified the living/dead state of fungal fruiting bodies as the major factor determining the associated beetle community. While, in soils, decomposing fungal biomass represents hotspots of microbial activity and diversity, due to a relatively high nitrogen content in an otherwise nitrogen-limited environment (Brabcová et al. 2016). The rate of mycelia decomposition is related to the carbon: nitrogen ratio. Saprotrophic fungi with hard fruiting bodies, similar to wood, and a high carbon: nitrogen ratio are decomposed slowly (Koide and Malcolm 2009, Fernandez and Koide 2014, Brabcová et al. 2018), providing a longer duration when they can serve as a habitat or growth substrate for other organisms.

We aimed to compare the symbiotic and decomposer community of naturally-occurring healthy living and rotten fruiting bodies of *F. fomentarius* using metagenomic and metatranscriptomic analyses to evaluate the role of different organisms during the time when living fruiting bodies produce fungal spores as well as during their decomposition. This allows us not only to identify the microbial community that co-exists with *F. fomentarius* and their functional traits but also to determine the transcriptionally active fraction of the community. We hypothesised that the fruiting bodies would serve as a growth substrate for mycophagous fungi and bacteria, as was previously observed when dead fungal mycelia were incubated in soil or litter (Brabcová et al. 2016, Brabcová et al. 2018). Due to the high abundance of *F. fomentarius* in Northern Hemisphere forests, this work will shed light on the dynamics of one of the major drivers of deadwood decomposition and carbon cycling in this environment.

## Methodology

### Sampling procedure

Ten fruiting bodies of *F. fomentarius* were sampled in the Žofínský Prales National Nature Reserve in the Czech Republic (48°39′57″N, 14°42′24″E) in September 2017. Fruiting bodies without signs of infection or damage were putatively considered healthy living while fruiting bodies with changed colour (darker and with neither a visible white line of growth nor fresh hymenia), visible infection, physical damage and no sign of fresh growth or spore production were putatively considered as dead and rotten. One putatively healthy living and one putatively rotten fruiting body was sampled from each of five selected decomposing tree trunks of *Fagus sylvatica*, the tree species dominant in this ecosystem (Baldrian et al. 2016). The mean size of the sampled fruiting bodies was 15-25 cm in diameter. The trees were chosen when both healthy living and rotten fruiting bodies were present on the same deadwood object with preference given to trees that were part of the longer term monitoring experiment. All trees were of a similar diameter (50–80 cm), decomposition stage (15–30 years of decomposition) and came from the same locality (50-300 m between deadwood). The fruiting body was cut from the tree using a sterile knife and all surface parts potentially associated with organisms that sedimented on the surfaces were removed. The entire inner part was shredded into fine chips, using a hand saw, and immediately flash-frozen in liquid nitrogen before being transported to the lab and stored at −80°C.

### Nucleic acid extraction and sequencing

Prior to DNA/RNA extraction, the sample was homogenized in liquid nitrogen by mortar and pestle. Total RNA was extracted, in triplicate, from 200 mg of sample material using the NucleoSpin RNA Plant kit (Macherey-Nagel) according to manufacturer's protocol after mixing with 900 µl of the RA1 buffer and shaking on FastPrep-24 (MP Biomedicals) at 6.5 m.s^−1^ twice for 20 s. Triplicates were pooled and treated with OneStep PCR Inhibitor Removal kit (Zymo Research). The DNA was removed using Turbo DNA-free kit (Invitrogen) and the efficiency of DNA removal was confirmed by PCR with the bacterial 515F (5′-GTGCCAGCM GCCGCGGTAA-3′) and 806R (5′-GGACTACHVGGGTWTCTAAT-3′) primers (Caporaso et al. 2012). Bacterial rRNA was depleted with the MICROBEexpress kit (Ambion). Eukaryotic, cytoplasmic and mitochondrial rRNA depletion as well as sequencing library preparation were done using the Trueseq Stranded Total RNA Gold kit (Illumina) following the manufacturer's instructions. The RNA quality was assessed using a 2100 Bioanalyzer (Agilent Technologies) after both DNAse treatment and library preparation. The libraries were sequenced on an Illumina HiSeq 2500 (2 × 250 bases) at Brigham Young University Sequencing Centre, USA.

Total genomic DNA was extracted from 200 mg of homogenized frozen material described above using the NucleoSpin Soil Kit (Macherey-Nagel, Germany) following the manufacturer's instructions. Briefly, cells were lysed using SL1 lysis buffer with Enhancer SX added prior to lysis. The samples were homogenized using a FastPrep-24 (MP Biomedicals, Santa Anna, United States) at 5 m.s^−1^ for 2 × 30 s. Two extractions per sample were performed and pooled. The metagenomic sequencing library was prepared using TrueSeq Nano DNA library preparation kit (Illumina) according to the manufacturer's protocol and sequenced in-house on the Illumina MiSeq (2 × 250 bp).

### Contig assembly and annotation of metagenomes and metatransciptomes

Trimmomatic 0.36 (Bolger et al. 2014) and FASTX-Toolkit (http://hannonlab.cshl.edu/fastx_toolkit/) were used to remove adaptor contamination, trim low-quality ends of reads and omit low quality (<30) reads or those shorter than 50 bp. rRNA reads were filtered out using the bbduk.sh (version 38.26) program from BBTools (https://sourceforge.net/projects/bbmap/) using a database of rRNA fragments from Silva (Quast et al. 2013). The combined metagenome assembly of all samples was performed using MEGAHIT 1.1.3 (Li et al. 2015), while the metatranscriptome assembly was done with Trinity 2.14.0 (Grabherr et al. 2011). Genes were predicted for both the metagenome and metatranscriptome assemblies using FragGeneScan (v.1.31, Rho et al. 2010).

A putative taxonomic identity was assigned to each gene by selecting the highest bit score after a BLAST search. Searches were performed against a database consisting of (1) an in-house assembled genome and transcriptome of a pure *F. fomentarius* culture isolated from a fruitbody collected in the study area (Supplementary File 1), (2) 2307 fungal genomes downloaded from the Joint Genome Institute's Mycocosm fungal genomics portal (https://mycocosm.jgi.doe.gov/, Grigoriev et al. 2014) and (3) the NCBI non-redundant proteins database (downloaded 07/10/2022). Due to the challenges of taxonomic identification in metagenomes, we are only reporting results at the family level or higher.

To obtain functional predictions, genes were annotated with KEGG Orthologs (KOs) by comparing the amino acid residues with the KOfam database (downloaded on October 2022, Aramaki et al. 2020) using HMM profiles and hmmsearch (HMMER v3.3.2, Eddy 2011). Matches were only considered for scores higher than the predefined thresholds and an e-value lower than 1e^−5^. Genes encoding the Carbohydrate-Active Enzymes (CAZymes) were annotated using the dbCAN HMM database V6 (Huang et al. 2018). The default settings were used for all tools.

### Analysis

Unless otherwise stated, all data was analysed using R 4.3.2 (R Core Team 2023). To increase reproducibility, where possible, package versions were managed with groundhog 3.1.1 (Simonsohn and Gruson 2023), set to the date 01/09/2023. During the analysis, we used the following CRAN packages and their dependencies: ggplot2 (Wickham 2016), RColorBrewer (Neuwirth 2022), pheatmap (Kolde 2019), ggrepel (Slowikowski 2021), cowplot (Wilke 2020), cluster (Maechler et al. 2022), NbClust (Charrad et al. 2014), shipunov (Shipunov et al. 2023), ggdendro (Vries and Ripley 2022), stringr (Wickham 2022), ggplotify (Yu 2021) and ggnewscale (Campitelli 2022). Groundhog can not currently provide version control for BioConductor packages, so the following package versions were used: Rsubread 2.14.2 (Liao et al. 2019).

The complete analysis script is available on Github (https://github.com/jasonbosch/Decomposing-Fomes-fomentarius-fruiting-bodies-represent-a-habitat-primarily-driven-by-Arthropoda). In brief, the assembled metagenomes and metatranscriptomes were imported and the number of reads matching each gene were counted using Rsubread's featureCounts function using the default settings. To facilitate comparison between different samples, all gene counts, in both the metagenomes and metatranscriptomes, were converted to transcripts per kilobase million (TPM).

In order to confirm the healthy living/rotten status of the samples, we determined the Euclidean distance between each sample. These samples were then plotted on a heatmap and analysed through Principal Components Analysis (PCA) with the function prcomp(). Furthermore, samples were clustered via hierachical clustering, based on the Euclidean distance, with hclust() using the method Ward.D2 which showed the best clustering structure. The clusters were bootstrapped with shipunov::Bclust() using 1000 iterations. Based on the combined results of the metagenomic and metatransciptomic analyses, the putative field classification was either retained or the sample was reassigned as either healthy living or rotten.

The community analysis and CAZyme expression was performed by aggregating the genes/transcripts by their attributes at different taxonomic levels. Metabolic potential was tested by converting KEGG modules into a logical expression and assessing the previously assigned KOs of genes with a TPM > 0.

## Results

### Classification of fruiting bodies

Fruiting body decomposition is a continuous process and visual assessment of the fruiting body appearance may not be fully reliable to identify the stage of decomposition of the fruiting body. Moreover, as material was sampled from inside the fruiting body, it was impossible to determine whether the outer appearance of the fruiting body corresponded to the status of its internal tissue while in the field. Supported by the metagenomic profile analysis (Fig. S1), we confirmed the classification of all putative healthy living samples (live fruiting bodies) as well as of the putative rotten samples H03, H16 and H20. However, the metagenomic analysis showed putative rotten samples H08 and H22 to cluster with the healthy living samples (Fig. S1). Sample H19 showed an intermediate metagenomic profile, but, due to the metatranscriptomic profile (the presence of *F. fomentarius* mRNA), was classified as healthy living. Sample H22 was excluded from further metatranscriptomic analysis due to the low number of reads obtained (<3500).

### Community composition of fruiting bodies

**Figure 1.**
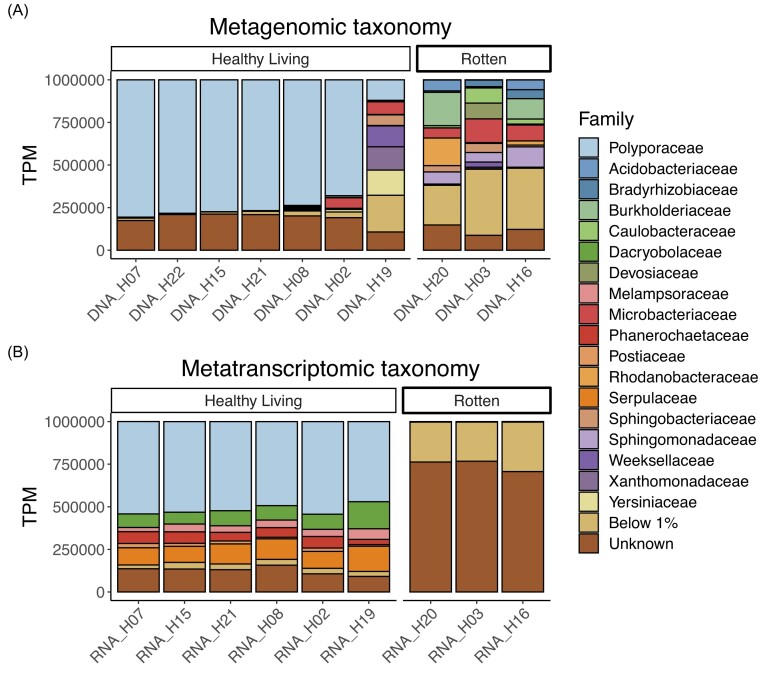
Composition and activity of the microbiome within *F. fomentarius* fruiting bodies. Family-level taxonomy assigned to the predicted genes in the (A) metagenomic and (B) metatranscriptomic sequencing of the fruiting bodies. The abundance of each gene was scaled using transcripts per kilobase million (TPM). Samples are ordered left-to-right in decreasing *F. fomentarius* TPM in the metagenome. The Polyporaceae family, to which *F. fomentarius* belongs, is listed at the top.

In the metagenome, we observed that ∼81% of the untransformed reads and ∼76% of the reads by TPM matched to the *F. fomentarius* genome in the samples from healthy living fruiting bodies (hereafter healthy living samples; Fig. 1). Less than 0.1% of reads matched to *F. fomentarius* in the rotten samples, indicating an absence of *F. fomentarius* DNA. Sample H19 showed a high abundance of bacterial sequences and a low abundance of *F. fomentarius*, indicating that it is in a transitional stage between healthy living and rotten. The rotten metagenome primarily consisted of reads assigned to bacteria (∼93% untransformed reads and ∼86% by TPM) and displayed higher taxon diversity than that of the healthy living samples; best hits of predicted genes in the metagenomes belonged to a median of 1151 families in rotten samples and 917 in the healthy living samples.

The majority of the rotten metagenomic community could be assigned to Proteobacteria with a much smaller contribution from Actinobacteria and Bacteroidetes (Fig. S2). In these samples the most-abundant families were Burkholderiaceae (10.7%), Microbacteriaceae (9.7%) and Sphingomonadaceae (8.2%). The abundance of Ascomycota was notably higher in the rotten samples, up to almost 6% in sample H20 (Fig. S3). In healthy living samples, the dominant family of Ascomycota was Magnaporthaceae but their relative share was low. Only sample H03 contained reads of one particularly dominant family: the Hypocreaceae. Samples H20 and H16 both had complex complements of Ascomycota with Hyaloscyphaceae, undefined Helotiales, Cephalothecaceae and Helotiaceae making up the largest share of the fungal reads.

The metatranscriptome displayed an overall similar composition to the metagenome; ∼67% of the untransformed reads and ∼51% of the reads by TPM matched to *F. fomentarius* in the healthy living samples (Fig. 1). In contrast to the metagenome, the metatranscriptome of the sample H19 closely matched that of the other healthy living samples. We also observe that most transcription in the rotten samples belonged to transcripts which could not be assigned to any particular taxon, indicating that the presence of various bacteria does not imply that they are all transcriptionally active. The origin of most rotten transcripts remained unknown even at the phylum level (Fig. S2).

### Functional potential of the bacterial community

In order to understand the metabolic potential in the bacterial community, we compared the KEGG Orthologs identified in the metagenome to those required for the complete metabolic pathways for methane, nitrogen and sulfur metabolism and photosynthesis as defined in KEGG modules (Kanehisa 2000). Due to the difficulties of matching metagenomic data to eukaryotic genes, we limited the analysis to bacterial genes only. Excluding nitrogen fixation, our analysis revealed that at least complete pathways could be reconstructed from the metagenomic data for formaldehyde assimilation, F420 biosynthesis, methanofuran biosynthesis, anoxygenic photosystem II and assimilatory sulfate reduction (Table S1). In several cases, complete pathways could be assigned to highly-abundant bacterial families (>1% of the TPM contributed by bacteria).

Regarding nitrogen metabolism, both the complete assimilatory and dissimilatory nitrate reduction pathways were present in both the healthy living and rotten fruiting bodies (Table S1). Dissimilatory nitrate reduction was potentially performed by Burkholderiaceae, Yersiniaceae, Comamonadaceae, Rhodanobacteraceae and Bradyrhizobiaceae. While the necessary genes to perform denitrification were detected in rotten fruiting bodies, this pathway was not complete in any of the highly-abundant bacterial families. The pathway for nitrogen fixation was not complete in either the healthy living or rotten fruiting body metagenome. A search for the individual nitrogenase subunits, *nifH* (K02588), *nifD* (K02586) and *nifK* (K02591), revealed no matches in the metatranscriptome and *nifD* transcripts in only four metagenomes (H03, H07, H20 and H21), all with a best hit to Xanthobacteraceae (Proteobacteria).

### CAZyme expression

**Figure 2.**
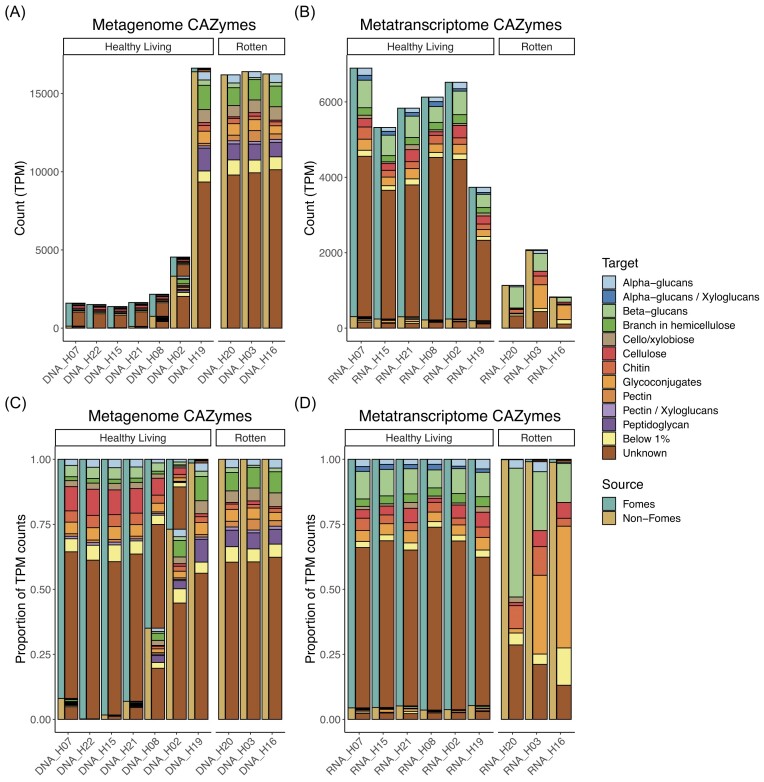
CAZymes in the healthy living and rotten fruiting bodies of *F. fomentarius*. Each sample is represented by two bars, the narrow, left bar shows whether the identified gene has a best-hit match to *F. fomentarius* or Non-*F. fomentarius* genomes while the thicker, right bar shows the CAZyme target substrate. CAZyme target substrates are shown as stacked barplots of (A & B) TPM and (C & D) TPM proportions for both the (A & C) metagenome and (B & D) metatranscriptome. Samples are ordered left-to-right in decreasing *F. fomentarius* TPM in the metagenome. Any CAZyme targets which accounted for less than 1% TPM were grouped together as “Below 1%.”

Carbohydrate-Active Enzymes (CAZymes) have a known role both in decomposition of biopolymers and are important for internal restructuring of *F. fomentarius* fruiting bodies (Bowman and Free 2006). Looking at the metagenomes, it can be observed that a greater number of CAZymes, by TPM, are present in rotten samples; this could reflect more compact bacterial genomes where CAZymes represent a larger percentage on the genome (Fig. 2A). However, the metatranscriptomics showed that the CAZymes of *Fomes* were highly expressed in its healthy living fruiting bodies. This was true even in the case of sample H19, which contained a large share of bacterial sequences (Fig. 2B), suggesting that *F. fomentarius* was more metabolically active than the bacteria. Based on the metagenomes, *F. fomentarius* showed a greater proportion of CAZymes targeting beta-glucans and cellulose compared to non-*F. fomentarius* CAZymes but lower proportions of CAZymes targeting hemicellulose branches and peptidoglycan (Fig. 2). However, the differences in the metatranscriptome were more dramatic. CAZyme expression by *F. fomentarius* broadly matched the gene complement while non-*F. fomentarius* expression in the rotten samples primarily targeted either beta-glucans or glycoconjugates (glycoproteins, glycolipids or proteoglycans). The proportion of CAZymes with an unknown target in rotten samples was also reduced, both in comparison to healthy living samples but also in comparison to the rotten metagenome.

**Figure 3.**
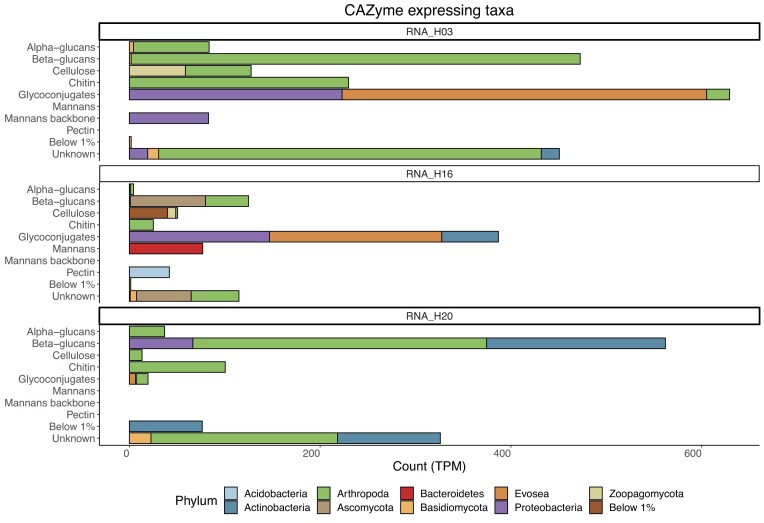
Expression of CAZymes targeting particular substrates in rotten *F. fomentarius* fruiting bodies by phylum. Each bar is coloured to show which phyla express transcripts targeting a particular biopolymer. Any taxa or targets which add up to less than 1% of the total TPM are grouped together as “Below 1%.”

Unsurprisingly, almost all CAZymes expressed in the healthy living sample belonged to *F. fomentarius;* many of them likely being involved in internal cell-wall remodelling processes but the transcription of genes required for deadwood decomposition was observed as well. CAZymes expressed in the rotten samples originated from a variety of phyla with the largest contribution from Arthropoda, Actinobacteria, Evosea and Proteobacteria (Fig. 3). We found that the majority (by TPM) of CAZymes expressed in all but one of the rotten samples, including all CAZymes targeting chitin and the majority (16/29 genes) that were predicted to target beta-glucans (Fig. 3, Table S2), were produced by Arthropoda. The Arthropoda chitinases were predicted to have the closest similarity to genes of three different families; Tenebrionidae (13 chitinases), Cerambycidae (1 chitinase) and Pyroglyphidae (1 chitinase). Additionally, one transcript belonging to Micromonosporaceae (Actinobacteria) was predicted to target both cellulose and chitin but this was neither widespread nor highly expressed.

Beta-glucanases were also produced by other arthropods (the Oppiidae and Acaridae mites and Chrysomelidae beetles), Actinobacteria (the families Micromonosporaceae and Streptomycetaceae) and Ascomycota (the families Cephalothecaceae and Chaetosphaeriaceae). However, except for Micromonosporaceae, chitinases were not detected and, except for Opiidae, they were not present in all the rotten samples.

The remaining taxa produced their own complements of CAZymes, often targeting specific substrates (Table S2). Acidobacteria produced a pectinase while Evosea, as well as several families of Proteobacteria, produced glycoconjugate-degrading enzymes. Chitinophagaceae (Bacteroidetes) and Myxococcales (Proteobacteria) produced CAZymes that targeted mannans and the mannan backbone respectively. Finally, Verrucomicrobia and Kickxellaceae produced CAZymes with cellulose as a putative target.

## Discussion

We aimed to compare the microbial communities found on healthy living and decomposing fruiting bodies of *F. fomentarius*. Using metagenomics and metatranscriptomics, we have analysed the genetic potential and taxonomic composition of both the complete and active fraction of the community. Special consideration was given to bacterial metabolic pathways, due to the ease of assembling bacterial transcripts, and CAZyme expression.

### Community composition of fruiting bodies

The three most abundant bacterial families in the rotten samples of *F. fomentarius* fruiting bodies were Burkholderiaceae (10.7%), Microbacteriaceae (9.7%) and Sphingomonadaceae (8.2%, Fig. 1). Members of the *Burkholderia* genus (Burkholderiaceae) are known to have interactions with multiple hosts, act as a soil saprotrophs and producers of anti-fungal compounds (Stoyanova et al. 2007). Intriguingly, *Burkholderia gladioli* is a symbiont associated with several Tenebrionidae beetles where it functions to protect their eggs from fungal colonisation (Kaltenpoth and Flórez 2020). The majority of CAZymes targeting chitin in the metatranscriptome had a best-hit match to Tenebrionidae sequences. Microbacteriaceae are likely present due to a role in deadwood decomposition. The genus *Curtobacterium*, from the family Microbacteriaceae, was identified as a globally-distributed degrader of organic matter (Chase et al. 2016) and Microbacteriaceae, together with Burkholderiaceae, were observed at high abundance in buried Norway Spruce wood blocks (Valette et al. 2023). Furthermore, Burkholderiaceae and Microbacteriaceae were both associated with the fungal genera *Penicillium* and *Trichoderma*, which may imply they have other funtions than merely deadwood decomposition (Valette et al. 2023). Sphingomonadaceae has previously been found, at relatively low levels, in the mycangia of the wood-burrowing ambrosia beetle *Platypus cylindrus* (Nones et al. 2021). Additionally, all three of these bacterial families were found at high abundance in either the adult or larval form of the black tinder fungus beetle *Bolitophagus reticulatus* (Tenebrionidae) collected from *F. fomentarius* fruiting bodies (Kaczmarczyk-Ziemba et al. 2019).

Given the long lifespan of *F. fomentarius* fruiting bodies, one would expect them to have strong anti-microbial protections and few bacteria present in the metagenome. Setting aside sample H19 due to its transitional state, bacteria comprised 0.03–11.6% of the TPM metagenome of healthy living *F. fomentarius* fruiting bodies. In the six metagenomes where *F. fomentarius* was dominant, only Microbacteriaceae (1.2%) was present at greater than 1% relative abundance. The next most abundant bacterial families were Yersiniaceae (0.3%) and Burkholderiaceae (0.3%). Both Microbacteriaceae and Burkholderiaceae were also present in the rotten *F. fomentarius* samples.

Regarding the Ascomycota identified on the *F. fomentarius* fruiting bodies, there are low levels of Magnaporthaceae in all the healthy living samples. While many Magnaporthaceae are known as plant pathogens, they also include saprotrophic fungi, such as the genera *Plagiosphaera* (Song et al. 2019) and *Muraeriata* (Huhndorf et al. 2008). However, the exact taxonomic position of these genera is in question (Feng et al. 2021). In the rotten samples, the most abundant families belong to three orders: Hypocreales, Helotiales and Sordariales. Hypocreaceae (Hypocreales) are known to act as saprotrophic (Mihál et al. 2007, Kepler et al. 2017) mycoparasites (Kepler et al. 2017) and have been observed on fruiting bodies of other polypores (Mihál et al. 2007). The families Hyaloscyphaceae and Helotiaceae, as well as some taxa undefined at the family level, all belong to the saprotrophic Helotiales order. Helotiales have previously been observed at high relative abundance in deadwood (Brabcová et al. 2022) and increasing in abundance in the soil after tree dieback due to bark beetle infestation (Štursová et al. 2014). The Helotiales were also the second most abundant order in the healthy living samples. The family Cephalothecaceae was at its highest abundance in sample H19 and was a member of the Sordariales order which are known to inhabit soil, wood and dung (Zhang et al. 2006). Together, this suggests that some of the detected taxa are either mycoparasitic, notably Hypocreaceae, or incidentally detected due to their close proximity as fellow deadwood-degrading fungi.

### Functional potential of the bacterial community

We were particularly interested in nitrogen metabolism as the deadwood environment is nitrogen-scarce in comparison to the fungal biomass and nitrogen content has been shown to be an indicator of the rate of mycelial decomposition (Koide and Malcolm 2009, Fernandez and Koide 2014, Brabcová et al. 2018). As in deadwood bacterial communities (Tláskal et al. 2021), both assimilatory and dissimilatory nitrate reduction pathways were present (Table S1). However, we found no evidence for potential nitrogen fixation in the fruiting body metagenomes or metatranscriptomes in either the healthy living or rotten fruiting bodies. This was intriguing as deadwood bacteria are best positioned to colonise the dead fruiting body and include nitrogen fixing bacteria that associate with wood-decay fungi (Hoppe et al. 2014, Bellenger et al. 2020, Gómez-Brandón et al. 2020, Tláskal et al. 2021).

The difference in nitrogen fixation capabilities between the bacteria inhabiting the deadwood and those which colonise the decaying *F. fomentarius* could have multiple causes and it's not yet possible to distinguish between them. Nitrogen fixation is inhibited by oxygen, and, while deadwood is anaerobic (Covey et al. 2016), the oxygen levels in *F. fomentarius* fruiting bodies may select against nitrogen fixing bacteria. Previous studies have noted nitrogen-fixing bacteria in other fruiting bodies (Gohar et al. 2020, Pent et al. 2020, Ren et al. 2022) and there is no reason to suspect that *F. fomentarius* represents a dramatically more aerobic environment. It should be noted that Gohar et al. (2020) did not examine any polypores and their longest-lived fruiting bodies only survived a month before decay. *F. fomentarius* fruiting bodies may survive for several years and this could result in very different selective pressures than experienced by shorter-lived species. However, the previously-mentioned studies identified nitrogen fixing bacteria in fruiting bodies by taxonomy and not by identifying nitrogen fixation genes. It's possible that the fruiting body communities do not require nitrogen fixing capabilities even though closely-related taxa are capable of performing this ecological role.

The simplest explanation is that the bacterial community is derived from the deadwood bacterial community but undergoes environmental filtering, in this case against nitrogen-fixing bacteria. The bacterial community of fruiting bodies of soil fungi show a large overlap with the soil (Pent et al. 2017, Liu et al. 2018, Pent et al. 2020) or deadwood (Ren et al. 2022) bacterial communities, with selection determined by the chemistry of the fruiting body (Pent et al. 2020). The nitrogen content of fungal fruiting bodies is much higher than that of deadwood (Baldrian et al. 2016, Brabcová et al. 2018), which might mean that there is no need for energy-intensive, nitrogen fixation on fruiting bodies. Alternatively, many beetles are known to live in *F. fomentarius* fruiting bodies (Thunes 1994, Økland 2002, Friess et al. 2019) and are known to transfer fungi to new environments (Eskalen et al. 2013, Seibold et al. 2019). Under this scenario, the lack of nitrogen fixation is not due to environmental filtering but because the bacterial community is derived from the beetle inhabitants of the deadwood. This hypothesis may be supported by the fact that the most abundant bacterial families all occur in insect hosts, including those collected from *F. fomentarius* fruiting bodies (Kaczmarczyk-Ziemba et al. 2019, Kaltenpoth and Flórez 2020, Nones et al. 2021) and is congruent with research showing that wood-boring beetles introduce beetle‐associated fungi to deadwood (Skelton et al. 2019). Future research should attempt to distinguish between these two possibilities.

We identified the pathway for methanofuran biosynthesis, necessary for methanogenesis, in the bacterial metagenomes of both the healthy living and rotten fruiting bodies (Table S1). However, despite *F. fomentarius* fruiting bodies being identified as a source of methane emissions (Mukhin and Voronin 2008, 2009), no complete methanogenesis pathways were identified. This result held even when analysing all KOs simultaneously, regardless of taxonomy or condition. This suggests that the methanogenesis pathway is truly absent as it can not be completed by any combination of genes sequenced in the metagenome.

### CAZyme expression

All the substrates targeted by CAZymes in the rotten metatranscriptomes are major components of fungal cell walls. In general, glycoproteins can comprise up to 50% of the mass of fungal cell walls (Bowman and Free 2006) while beta-glucans and chitin contribute between 20%–69% and 5%–6% of the fruiting body mass respectively (Kolundžić et al. 2016, Sari et al. 2017, Kalitukha and Sari 2019, Pylkkänen et al. 2023). CAZymes targeting mannans were only detected in sample H16 (Fig. 3) but this too is a known component of fungal cell walls, although accounting for less than 1% of the dry mass (Kalitukha and Sari 2019). This is in line with expectations that the rotten fruiting body community will feed on the fungal structures that remain after the death of *F. fomentarius*.

The majority of CAZymes exclusively targeting chitin were produced by arthropods, specifically the orders Coleoptera (beetles) and Sarcoptiformes (mites; Fig. 3 & Table S2). Several members of both Coleoptera (Sinha 1966, Filipiak and Weiner 2014, McFarlane et al. 2021) and Sarcoptiformes (Schneider and Maraun 2005, Schneider et al. 2005, Koukol et al. 2009, Naegele et al. 2013) are known to feed on fungi. However, it is difficult to confidently assign taxonomic identity to Arthropoda metagenomic sequences due to the lack of available genomes, especially for Coleoptera where fewer than 0.01% of species have genomes available (Feron and Waterhouse 2022).

Chitin is not only a component of fungal cell walls but also a major building block of the Arthropoda exoskeleton. As many Arthropod species are known to inhabit, or associate with, *F. fomentarius* fruiting bodies (Thunes 1994, Økland 2002, Friess et al. 2019), it is possible that the Arthropoda CAZymes targeting chitin are intended for restructuring the exoskeleton and not digesting fungal cell walls. We believe this is unlikely as many arthropods are known to feed on fungi, CAZymes targeting beta-glucans were also best matches to Arthropoda sequences and no other taxa expressed CAZymes targeting chitin. The amount of chitin in the fruiting bodies was not quantified and it is possible that the *F. fomentarius* chitin had already been degraded. This, again, underlines the importance of having a timeline of fruiting body decomposition.

### Taxa involved in *F. fomentarius* fruiting body decomposition

Our metatranscriptome analysis suffered from the difficulties with rRNA removal (median rRNA content of 75%) and, as a result, a relatively shallow sequencing depth was achieved for mRNA and may have resulted in some taxa being omitted. While the high abundance of Arthropoda sequences implies that they are the dominant fungal decomposers, members of at least two microbial families may also play a role in *F. fomentarius* decomposition. The first is Micromonosporaceae (Actinobacteria) which secreted CAZymes that targeted both beta glucans and cellulose/chitin. The best-match genus was *Actinoplanes* and *Actinoplanes missouriensis* has been shown to be attracted to fungal spores (Arora 1986) and to have anti-fungal capabilities due to the production of chitinases (El-Tarabily 2003). Micromonosporaceae transcripts were not present in all samples and likely plays only a minor role or is present at only a certain stage of the decomposition process. The second family is Physaraceae (slime mould from the phylum Evosea). Although Physaraceae only expressed glycoconjugate-degrading enzymes, this is the most highly expressed CAZyme by genus (Table S2) and might suggest that Physaraceae decomposes only the glycoconjugates in *F. fomentarius* cell walls. Furthermore, several Physaraceae members have been previously reported to be mycophagous (Howard and Currie 1932a,b), with *Physarum polycephalum* and *Physarum tenerum* plasmodia specifically being identified as feeding on *F. fomentarius* mycelia (Howard and Currie 1932b).

Previous results identified specific microbial communities which established on dead fungal material (Brabcová et al. 2016) and we expected to find similar microbial, or functional, communities responsible for decomposing the dead fruiting bodies. Contrary to our expectations, almost all CAZyme transcripts targeting chitin came from Arthropoda. The community analysis in Brabcová et al. (2016) was conducted using 16S and ITS sequencing which would not have detected the involvement of insects even if they were the dominant decomposers. This suggests that future studies of fungal decomposition should make use of metatranscriptomics to identify the active community members with fewer taxonomic biases.

Alternatively, there may be qualitative differences between fungal decomposition in the soil as compared to fruiting bodies on deadwood. There is a rich community of bacteria and fungi in the soils which may rapidly colonise dead fungal material (Brabcová et al. 2016, López-Mondéjar et al. 2018, 2020). In contrast, the fruiting bodies of *F. fomentarius* are generally not in contact with the soil but with the deadwood itself—whose community, perhaps, does not have the same capacity for decomposition—and the microbe-scarce air. In this situation, it's possible that insects will find and feed on the fruiting bodies before microbes have an opportunity to colonise the dead fruiting body. There may be ecological value in comparing the decomposition of fungal material dependent on whether it has contact with the soil or not and when insect access is restricted or not. Potentially, decomposition of fruiting bodies with soil contact is dominated by microbes while aerial fruiting bodies are predominantly decomposed by Arthropoda.

### Limitations

We acknowledge that this work has some limitations, such as chronology, sample size and taxonomic identification. While the fruiting bodies were sampled as healthy living and highly decomposed and sorted into a binary classification of either healthy living or rotten, in reality they exhibited a gradient of decreasing *F. fomentarius* abundance and an increase in the abundance of bacteria and Ascomycota when going from healthy living to rotten samples of *F. fomentarius* fruiting bodies. Given the limited data available, we can not speculate on the timeline of fruiting body decomposition nor whether it is made up of distinct stages. Such questions must be answered with further work on more extensive set of fungal fruiting bodies. Despite the small sample size, the differences between healthy living and rotten samples were clear (e.g. *F. fomentarius* in the healthy living samples accounts for ∼80% of the metagenomic reads compared to <1% in the rotten samples.) and are consistent within classes. There is no reason to think that larger sample sizes would have changed our broad conclusions. We present our results here at the family level due to the difficulties of confidently assigning a taxonomy to metagenomic sequences, particularly for Arthropoda. This is a problem for all work relying on sequence databases and is beyond our control. More sensitive techniques will be necessary to follow up on this work but giving the current technological and database limitations, our results serve as a useful guide for future research.

## Conclusion

During the transition from live to rotten fruiting bodies, the microbiome of *F. fomentarius* fruiting bodies changes to become primarily bacteria-dominated. Despite being physically most closely associated with the adjacent deadwood, the bacterial community of *F. fomentarius* fruiting bodies did not appear to possess the ability to fix nitrogen. We recorded a greater abundance of CAZymes targeting beta-glucans and cellulose from the *F. fomentarius*-derived DNA while bacteria-derived DNA had a greater abundance of CAZymes targeting hemicellulose and peptidoglycan. However, while metatranscriptomics showed that *F. fomentarius* CAZyme expression broadly matched its genomic potential, CAZyme transcripts expressed in rotten fruiting bodies were enriched for enzymes targeting components of the fungal cell wall, namely beta-glucans, glycoconjugates and chitin. The majority of these CAZymes best matched to Arthropoda sequences, including almost all CAZymes which target chitin. Given prior knowledge of mycophagous beetles and the fact that the Arthropoda CAZymes also target beta-glucans, it appears that *Arthropoda* are the likely primary decomposers of *F. fomentarius* fruiting bodies.

## Supplementary Material

fiae044_Supplemental_Files

## Data Availability

All sequencing data is available from the NCBI as BioProject PRJNA1037776 (http://www.ncbi.nlm.nih.gov/bioproject/1037776). Scripts to reproduce the analysis are available on Github (https://github.com/jasonbosch/Decomposing-Fomes-fomentarius-fruiting-bodies-represent-a-habitat-primarily-driven-by-Arthropoda) and all the processed files used in the script can be downloaded from Zenodo (https://doi.org/10.5281/zenodo.10081831).
