# Correction to: decomposition of *fomes fomentarius* fruiting bodies – transition of healthy living fungus into a decayed bacteria-rich habitat is primarily driven by Arthropoda

**DOI:** 10.1093/femsec/fiae077

**Published:** 2024-05-22

## Abstract

Analysis of microbial communities in tiny water droplets enclosed in oil allows to infer underlying community assembly processes of the taxonomic core community.

This is a correction to: Jason Bosch, Priscila Thiago Dobbler, Tomáš Větrovský, Vojtěch Tláskal, Petr Baldrian, Vendula Brabcová, Decomposition of *Fomes fomentarius* fruiting bodies—transition of healthy living fungus into a decayed bacteria-rich habitat is primarily driven by Arthropoda, *FEMS Microbiology Ecology*, Volume 100, Issue 5, May 2024, https://doi.org/10.1093/femsec/fiae044

In the version originally published, the tested fungus incorrectly read: “*Fomes fomentatius”* in the article title. This has been corrected in the article to read: *“Fomes fomentarius”*.

