# Supplementary material for: Decomposition of Fomes fomentarius fruiting bodies – transition of healthy living fungus into a decayed bacteria-rich habitat is primarily driven by Arthropoda
Source: FEMS Microbiol Ecol. 2024 Mar 29;100(5):fiae044. doi: 10.1093/femsec/fiae044 (PMC11030162; doi:10.1093/femsec/fiae044)
Supplement: fiae044_Supplemental_Files [file fiae044_supplemental_files.zip › Supplemental_File_1.docx]

### Methods for the in-house *Fomes fomentarius* genome and transcriptome

**Strain Isolation and Culturing**

A *F. fomentarius* fruiting body was collected in Žofínský Prales National Nature Reserve from tree no 106 (Tláskal *et al.* 2021), in September 2021, and isolated on ME (malt extract) agar supplemented with streptomycin to prevent bacterial growth. The pure culture was frequently replated on ME agar plates and cultured for DNA and RNA extraction in 2% malt extract liquid medium for one week. Actively growing culture was collected and frozen at -80°C until DNA and RNA extraction.

**DNA extraction and sequencing**

The DNA of a pure strain of *F. fomentarius* was extracted using the SK method (Sagova-Mareckova *et al.* 2008). Extracted DNA was used for long- and short-read sequencing. The SQK-LSK108 ligation kit (Oxford Nanopore Technologies) was used to prepare a long-read sequencing library according to the manufacturer’s instructions. The library was loaded onto a Nanopore flow-cell version FLO-MIN106 for a 48 h sequencing run. Sequencing adapters for short-read sequencing were ligated by the TruSeq DNA PCR-Free Library Prep Kit (Illumina Inc., United States). The ligated library was sequenced on an Illumina HiSeq 2500 (2 × 250 bp) at Brigham Young University Sequencing Centre, USA.

**Genome assembly**

The obtained FAST5 long reads were basecalled into FASTQ with local Albacore 2.3.1 (available via ONT community site: [https://community.nanoporetech.com](https://community.nanoporetech.com/)) with a minimal quality threshold of 7. Passed reads were scanned for remaining adapters, which were trimmed with Porechop 0.2.32 (<https://github.com/rrwick/Porechop>) using –end_threshold 75, –min_split_read_size 300 and –discard_middle settings. *F. fomentarius* *de novo* hybrid genome assembly was performed using SPAdes 3.12.0 (Bankevich *et al.* 2012) with paired-end short reads and long reads using –nanopore settings and k-mer sizes 21, 33, 55, 77, 99 and 127. The final assembly had 16X genome coverage.

**RNA extraction and sequencing**

The RNA from the pure culture of *F. fomentarius* was extracted using a NucleoSpin RNA Plant kit (Macherey-Nagel) according to manufacturer’s protocol after mixing with 900 μl of the RA1 buffer and shaking on FastPrep-24 (MP Biomedicals) at 6.5 ms−1 twice for 20 s. Extracted RNA was treated as described previously (Tláskal *et al.* 2021) with OneStep PCR Inhibitor Removal kit (Zymo Research), DNA was removed using DNA-free DNA Removal Kit (Thermo Fisher Scientific). The efficiency of DNA removal was confirmed by the negative PCR results with the bacterial primers 515F and 806R (Caporaso *et al.* 2012). RNA quality was assessed using a 2100 Bioanalyzer (Agilent Technologies). rRNA in RNA samples was reduced using a Ribo-Zero rRNA Removal Kit Human/Mouse/Rat (Illumina). The efficiency of the removal was checked using a 2100 Bioanalyzer and removal was repeated when necessary. Reverse transcription was performed with SuperScript III (Thermo Fisher Scientific). Libraries for high-throughput sequencing were prepared using the ScriptSeq v2 RNA-Seq Library Preparation Kit (Illumina) according to the manufacturer’s instructions with a final 14 cycles of amplification by FailSafe PCR Enzyme (Lucigen).

**Transcriptome assembly**

Transcriptome assembly was performed using Trimmomatic 0.36 (Bolger, Lohse and Usadel 2014) and FASTX-Toolkit (<http://hannonlab.cshl.edu/fastx_toolkit/>) which were used to remove adaptor contamination, trim low-quality ends of reads and omit reads with overall low quality (<30); sequences shorter than 50 bp were omitted. mRNA reads were filtered from the data using the bbduk.sh 38.26 program in BBTools (<https://sourceforge.net/projects/bbmap/>). Transcriptome assembly was performed using Trinity v2.12.0 with parameters: --seqType fq --max_memory 128G --CPU 256.

**Data Availability**

This Whole Genome Shotgun project has been deposited at DDBJ/ENA/GenBank under the accession JAZHQO000000000. The version described in this paper is version JAZHQO010000000.

This Transcriptome Shotgun Assembly project has been deposited at DDBJ/EMBL/GenBank under the accession GKRL00000000. The version described in this paper is the first version, GKRL01000000.

### References

Bankevich A, Nurk S, Antipov D *et al.* SPAdes: A New Genome Assembly Algorithm and Its Applications to Single-Cell Sequencing. *Journal of Computational Biology* 2012;**19**:455–77, DOI: 10.1089/cmb.2012.0021.

Bolger AM, Lohse M, Usadel B. Trimmomatic: a flexible trimmer for Illumina sequence data. *Bioinformatics* 2014;**30**:2114–20, DOI: 10.1093/bioinformatics/btu170.

Caporaso JG, Lauber CL, Walters WA *et al.* Ultra-high-throughput microbial community analysis on the Illumina HiSeq and MiSeq platforms. *ISME J* 2012;**6**:1621–4, DOI: 10.1038/ismej.2012.8.

Sagova-Mareckova M, Cermak L, Novotna J *et al.* Innovative Methods for Soil DNA Purification Tested in Soils with Widely Differing Characteristics. *Appl Environ Microbiol* 2008;**74**:2902–7, DOI: 10.1128/AEM.02161-07.

Tláskal V, Brabcová V, Větrovský T *et al.* Metagenomes, metatranscriptomes and microbiomes of naturally decomposing deadwood. *Sci Data* 2021;**8**:198, DOI: 10.1038/s41597-021-00987-8.
