# Supplementary figures and images for: Decomposition of Fomes fomentarius fruiting bodies – transition of healthy living fungus into a decayed bacteria-rich habitat is primarily driven by Arthropoda
Source: FEMS Microbiol Ecol. 2024 Mar 29;100(5):fiae044. doi: 10.1093/femsec/fiae044 (PMC11030162; doi:10.1093/femsec/fiae044)

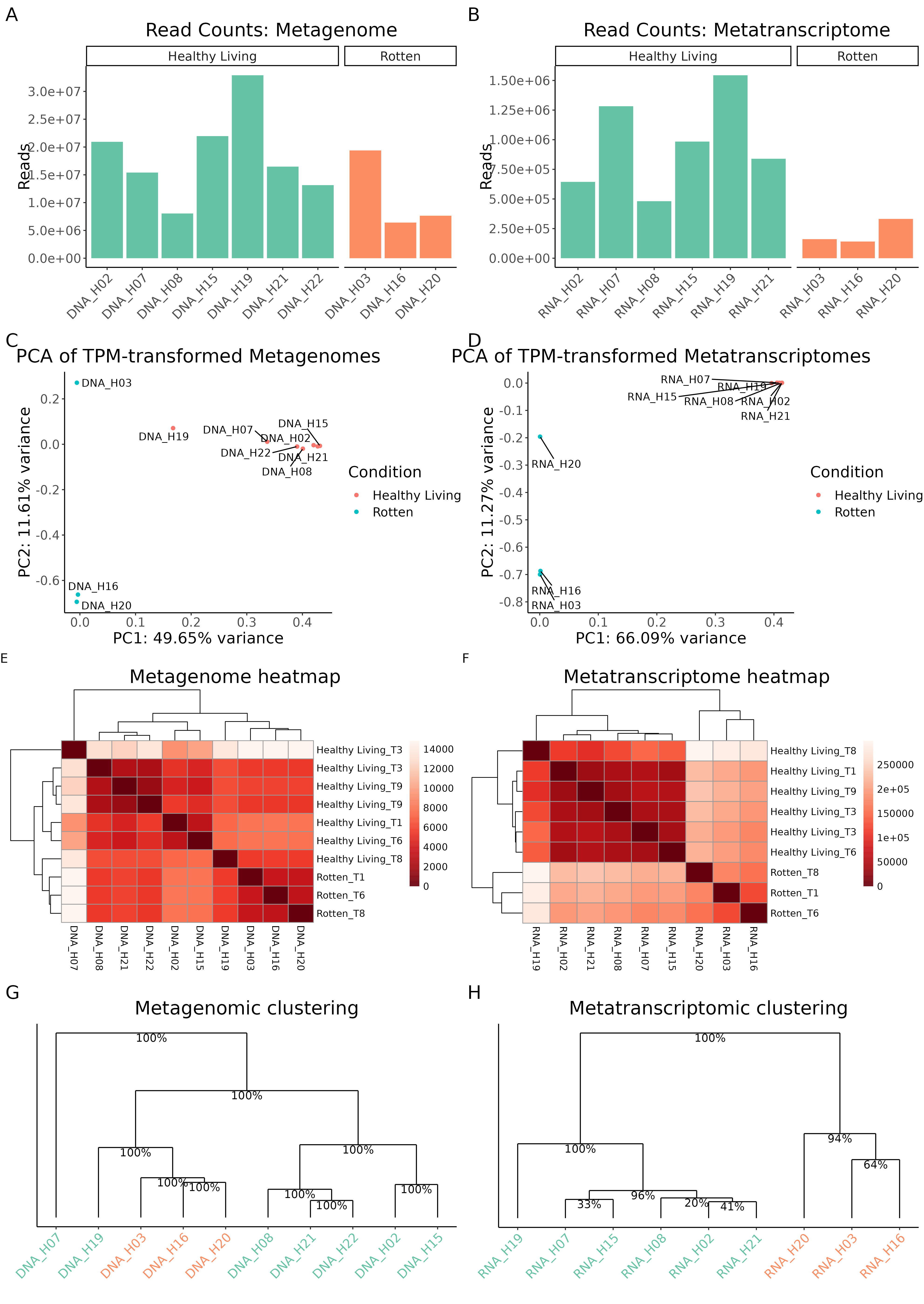

Supplement: fiae044_Supplemental_Files [file fiae044_supplemental_files.zip › Supp data Figure_S1.jpg]

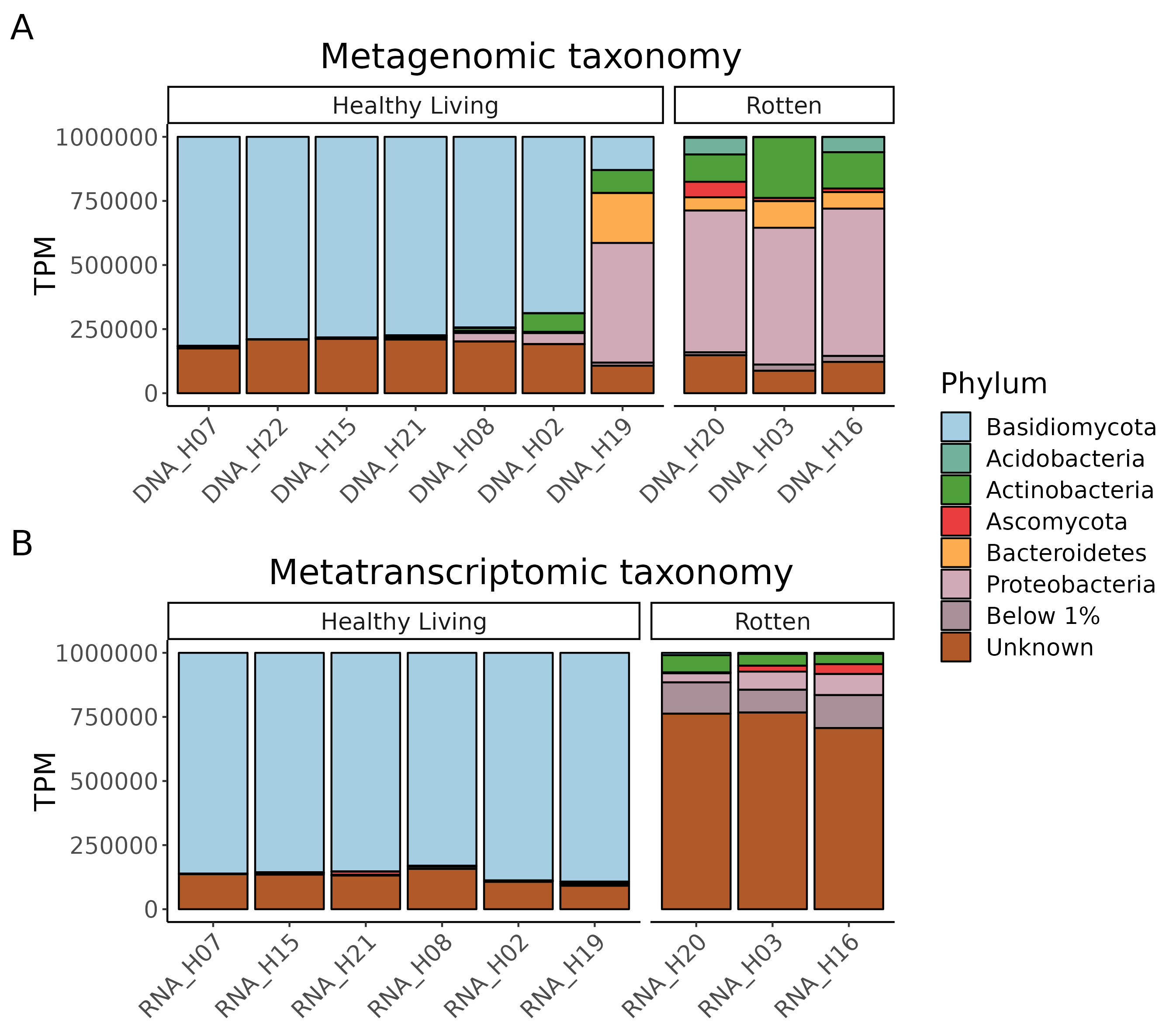

Supplement: fiae044_Supplemental_Files [file fiae044_supplemental_files.zip › supp data Figure_S2.jpg]

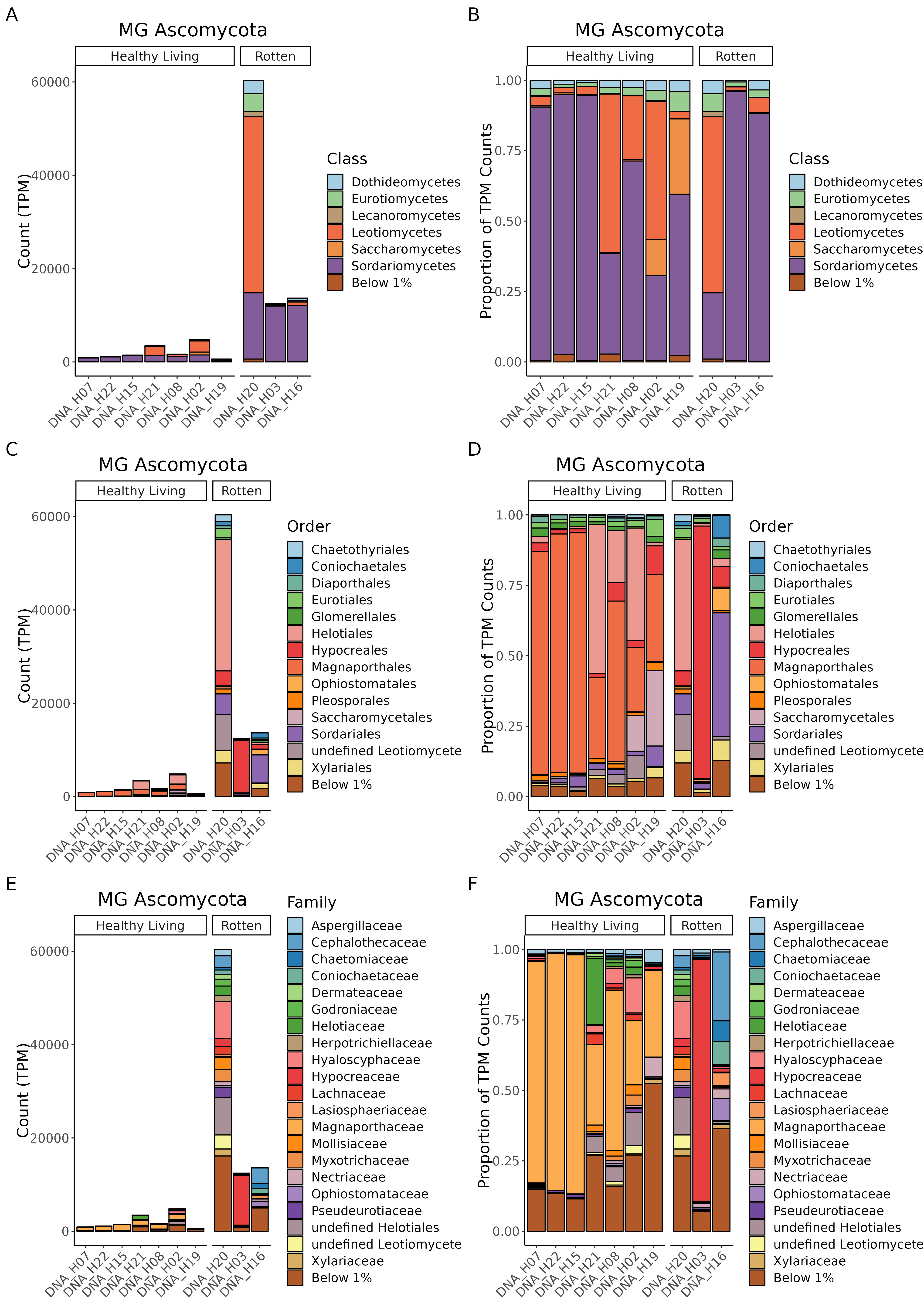

Supplement: fiae044_Supplemental_Files [file fiae044_supplemental_files.zip › supp data Figure_S3.jpg]
